# Defunct brain stem cardiovascular regulation underlies cardiovascular collapse associated with methamphetamine intoxication

**DOI:** 10.1186/1423-0127-19-16

**Published:** 2012-02-07

**Authors:** Faith CH Li, JC Yen, Samuel HH Chan, Alice YW Chang

**Affiliations:** 1 Center for Translational Research in Biomedical Sciences, Kaohsiung Chang Gung Memorial Hospital, Kaohsiung 833, Taiwan, Republic of China; 2 Institute of Pharmacology, National Yang Ming University, Taipei 112, Taiwan, Republic of China

## Abstract

**Background:**

Intoxication from the psychostimulant methamphetamine (METH) because of cardiovascular collapse is a common cause of death within the abuse population. For obvious reasons, the heart has been taken as the primary target for this METH-induced toxicity. The demonstration that failure of brain stem cardiovascular regulation, rather than the heart, holds the key to cardiovascular collapse induced by the pesticide mevinphos implicates another potential underlying mechanism. The present study evaluated the hypothesis that METH effects acute cardiovascular depression by dampening the functional integrity of baroreflex via an action on brain stem nuclei that are associated with this homeostatic mechanism.

**Methods:**

The distribution of METH in brain and heart on intravenous administration in male Sprague-Dawley rats, and the resultant changes in arterial pressure (AP), heart rate (HR) and indices for baroreflex-mediated sympathetic vasomotor tone and cardiac responses were evaluated, alongside survival rate and time.

**Results:**

Intravenous administration of METH (12 or 24 mg/kg) resulted in a time-dependent and dose-dependent distribution of the psychostimulant in brain and heart. The distribution of METH to neural substrates associated with brain stem cardiovascular regulation was significantly larger than brain targets for its neurological and psychological effects; the concentration of METH in cardiac tissues was the lowest among all tissues studied. In animals that succumbed to METH, the baroreflex-mediated sympathetic vasomotor tone and cardiac response were defunct, concomitant with cessation of AP and HR. On the other hand, although depressed, those two indices in animals that survived were maintained, alongside sustainable AP and HR. Linear regression analysis further revealed that the degree of dampening of brain stem cardiovascular regulation was positively and significantly correlated with the concentration of METH in key neural substrate involved in this homeostatic mechanism.

**Conclusions:**

We conclude that on intravenous administration, METH exhibits a preferential distribution to brain stem nuclei that are associated with cardiovascular regulation. We further found that the concentration of METH in those brain stem sites dictates the extent that baroreflex-mediated sympathetic vasomotor tone and cardiac responses are compromised, which in turn determines survival or fatality because of cardiovascular collapse.

## Background

The psychostimulant methamphetamine (METH; N-methyl-O-phenylisopropylamine) is among the most popular illegal drugs worldwide [1-4]. Based on records between 1998 and 2001 from the Ministry of Justice, Taiwan, it has been reported that this schedule II controlled drug is being used by approximately 79.3% of drug offenders in Taiwan [5]. Because METH enhances alertness, concentration, energy, euphoria and libido, it has become a well-known drug of abuse that leads to severe societal and economical consequences [6,7]. The best known pharmacological effects of METH are well documented to be associated with brain sites that are involved in the reward system [8-11] and neurodegenerative disease [11-13]. As such, nucleus accumbens (NACC), globus pallidas (GPi), caudate nucleus (Cd), substantia nigra (SN) and ventral tegmental nucleus (VTA) are among the most often mentioned brain targets for METH-induced psychological or neurological responses [8-13].

METH intoxication is a common cause of death within the abuse population [14,15]. In particular, severe hypotension and bradycardia are often observed in patients who exhibit acute METH intoxication [1,15,16], with 100% mortality despite intensive care in a hospital setting [1,17]. For obvious reasons, the heart has been taken as the primary target for METH-induced cardiovascular toxicity. Thus, atrioventricular arrhythmia and myocardial ischemia reportedly contribute to METH-induced fatality [18]. Of note is that our laboratory [19] demonstrated recently that failure of brain stem cardiovascular regulation, rather than the heart, holds the key to cardiovascular collapse induced by the pesticide mevinphos. This suggests that another target for METH-induced cardiovascular toxicity may be brain stem nuclei, including nucleus tractus solitarii (NTS) and rostral or caudal ventrolateral medulla (RVLM or CVLM) that are associated with the baroreflex, which is responsible for the maintenance of stable blood pressure and heart rate [20].

The present study evaluated the hypothesis that METH effects acute cardiovascular depression by dampening the functional integrity of baroreflex via an action on brain stem nuclei that are associated with this homeostatic mechanism. Based on evaluations of distribution of METH in brain and heart on intravenous administration, baroreflex responses and survival rate and time, this hypothesis was validated.

## Methods

Adult male Sprague-Dawley rats (275-315 g, n = 39) purchased from the Experimental Animal Center of the National Science Council and BioLASCO, Taiwan, Republic of China were used. Rats were housed in an Association for Assessment and Accreditation of Laboratory Animal Care (AAALAC) International-accredited animal facility under temperature control (24-25°C) and 12-h light-dark cycle. Standard laboratory rat chow and tap water were available ad libitum. All experimental procedures carried out in this study were approved by the Institutional Animal Care and Use Committee of the Kaohsiung Chang Gung Memorial Hospital (CGMH891071). Efforts were made to reduce the number of animals used and to minimize animal suffering during the experiment.

### General preparation

Under an induction dose of pentobarbital sodium (50 mg/kg, i.p.), animals received preparatory surgery that included tracheal intubation and cannulation of the femoral artery and vein. During the recording session, which routinely commenced 60 min after the administration of pentobarbital sodium, animals received an intravenous infusion of propofol (20-25 mg/kg/h; Zeneca, Macclesfield, England), which provided satisfactory maintenance of anesthesia while preserving the capacity of central cardiovascular regulation [21]. Animals were allowed to breathe spontaneously with room air, and body temperature was maintained at 37°C by a heating pad.

### Evaluation of baroreflex responses

Arterial pressure (AP) signals recorded from the femoral artery were processed by an arterial blood pressure analyzer (APR31a; Notocord, Croissy-Sur-Seine, France) and heart rate (HR) was derived instantaneously from the systolic blood pressure (SBP) signals. To evaluate brain stem cardiovascular regulation, the SBP signals were subject simultaneously to on-line and real-time spectral analysis (SPA10a; Notocord). We were particularly interested in the low-frequency (LF; 0.25-0.8 Hz) component because it takes origin from RVLM [22], and its power density mirrors the prevalence of baroreflex-mediated sympathetic neurogenic vasomotor discharges that emanate from RVLM [23]. We also computed the baroreflex effectiveness index (BEI) based on online detection of spontaneous baroreflex sequences (BRS10a, Notocord), which were detected when SBP and pulse interval increased or decreased simultaneously [24]. As an index for baroreflex-mediated regulation of cardiac responses, BEI is defined as the total number of sequences divided by the total number of pressure ramps in each analysis zone.

### Intravenous administration of methamphetamine

METH was obtained from the Food and Drug Administration, Department of Health, Executive Yuan, Taipei, Taiwan. METH and saline (vehicle control) were administered intravenously. Temporal changes in pulsatile AP, mean AP (MAP), HR, power density of the LF component of SBP signals and BEI were routinely followed for 240 min after intravenous administration of METH in an on-line and real-time manner [25,26]. The survival time and survival rate within 240 min were also recorded.

### Collection of tissue samples from brain and heart

We routinely collected tissue samples from RVLM, CVLM, NTS, NACC, GPi, Cd, SN, VTA and heart at 20 or 240 min after the administration of METH or saline. Animals were killed with an overdose of pentobarbital sodium and neural tissues were collected bilaterally by micropunches according to their anatomical boundaries; cardiac tissues were collected directly from the left ventricle. The concentration of total proteins extracted from those tissue samples was determined by the BCA protein assay (Pierce, Rockford, IL, USA).

### Determination of METH in tissue samples

The concentration of METH in cell lysate extracted from RVLM, CVLM, NTS, NACC, GPi, Cd, SN, VTA or heart was determined according to the manufacturer's protocol of a commercial METH indirect enzyme-linked immunosorbent assay (ELISA) kit (Calbiotech, Spring Valley, CA, USA), in conjunction with spectrophotometric determination (Multiskan Spectrum, Thermo Fisher Scientific Waltham, MA, USA) of the absorbance of reaction solution at 450 nm [27,28].

### Statistical analysis

All values are expressed as mean ± SEM. The temporal effects of METH or saline treatments on MAP, HR, power density of the LF component of SBP signals, BEI or tissue METH concentration were assessed using one-way or two-way analysis of variance (ANOVA) with repeated measures, as appropriate, for group means. In both cases, Scheffé multiple-range test was used for post hoc comparison of individual means. Mortality was assessed with the Fisher Exact Test. Correlation between tissue METH concentration in RVLM and BEI or power density of LF component of SBP signals was determined by linear regression analysis. *P <*0.05 was considered to be statistically significant.

## Results

### Differential distribution of METH to brain and heart tissues after intravenous administration

**Figure 1:**
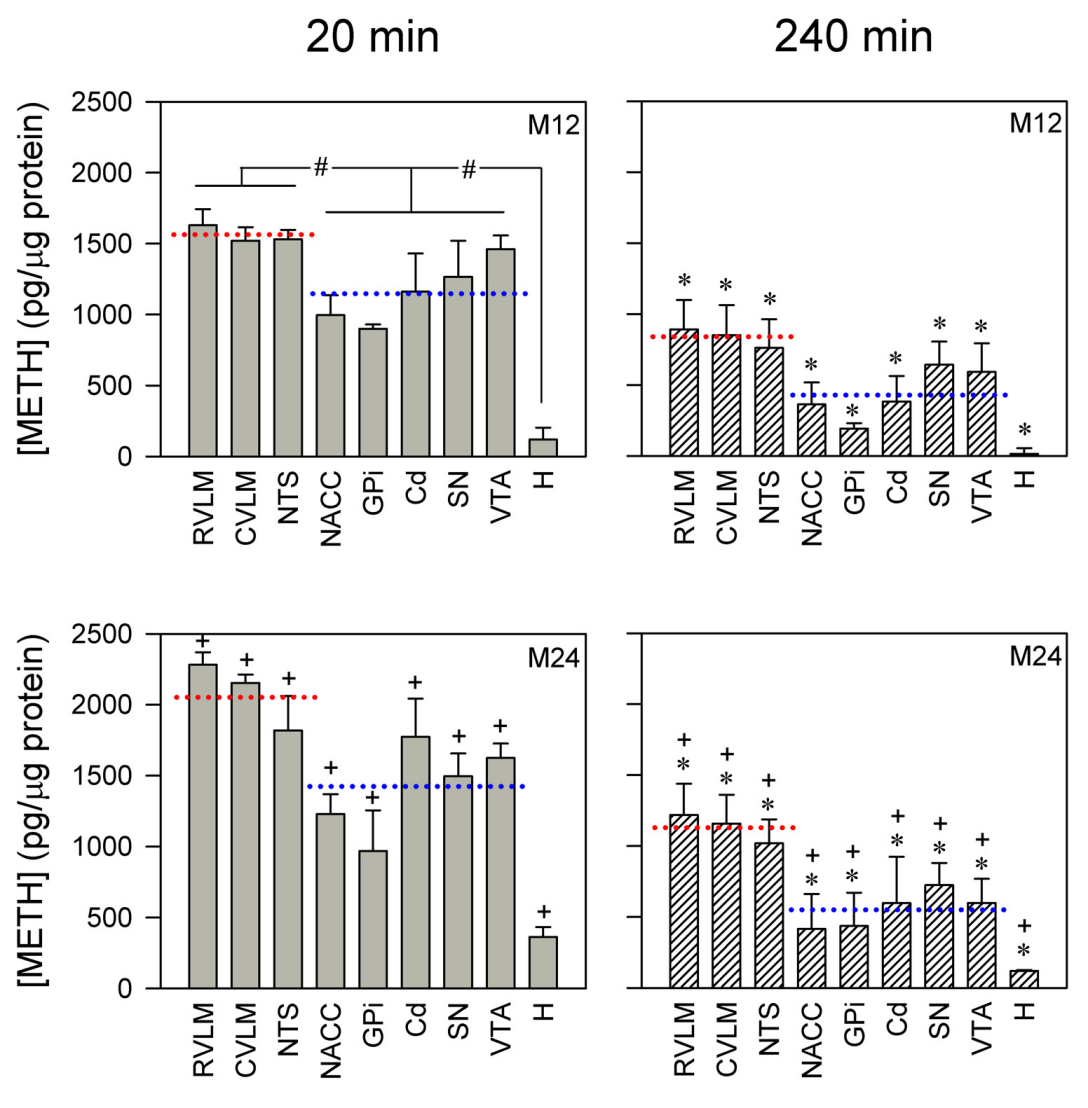
**Differential distribution of methamphetamine to brain and heart tissues after intravenous administration**. Changes in methamphetamine (METH) concentration in rostral ventrolateral medulla (RVLM), caudal ventrolateral medulla (CVLM), nucleus tractus solitarii (NTS), nucleus accumbens (NACC), globus pallidas (GPi), caudate nucleus (Cd), substantia nigra (SN), ventral tegmental area (VTA) or heart (H) collected from rats 20 min (gray bar) or 240 min (hatched bars) after intravenous administration of METH at 12 mg/kg (M12) or 24 mg/kg (M24). Values are mean ± SEM, n = 4-6 animals per experimental group. Red dotted line represents the mean value of METH concentration in RVLM, CVLM and NTS and blue dotted line represents the mean value of METH concentration in NACC, GPi, Cd, SN and VTA. Whereas^#^*P *< 0.05 versus heart tissue exists in all four subsets, it is only denoted in one subset for clarity. *P < 0.05 versus 20 min group at corresponding dose, and^+^*P *< 0.05 versus M12 group at corresponding time-points in the post hoc Scheffé multiple-range test.

Figure 1 shows that on intravenous administration, METH (12 or 24 mg/kg) exhibited a time-dependent distribution in brain and heart. While maintaining a dose-relationship, the concentration of METH in brain and heart tissues 20 min after administration of the two doses was approximately double that when measured at 240 min. Detailed analysis revealed two interesting observations. First, the distribution to the heart, a commonly mentioned target for cardiovascular effects of METH [1,29,30], was significantly less than brain tissues. Second, the concentration of METH was significantly higher in neural substrates associated with brain stem cardiovascular regulation (RVLM, CVLM or NTS) than brain targets for its psychological and neurological effects (NACC, GPi, Cd, SN or VTA).

### METH dose-dependently reduces survival

**Figure 2:**
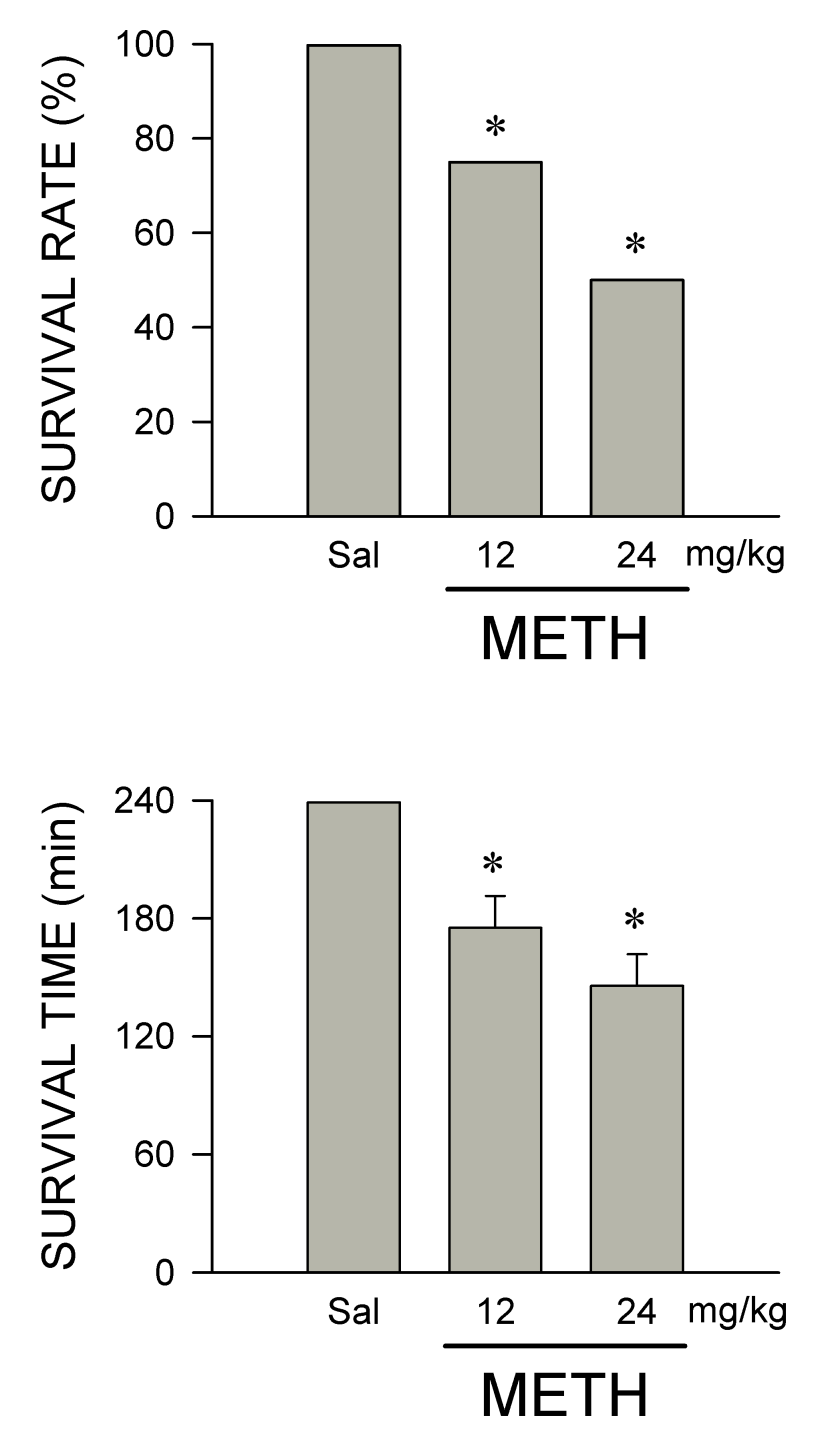
**METH dose-dependently reduces survival rate and survival time**. Dose-dependent changes in survival rate and survival time in rats that received intravenous administration of METH or saline (Sal, vehicle control). Values are mean ± SEM, n = 5-16 animals per experimental group at the beginning of the experiment. *P < 0.05 versus saline group in the Fisher Exact Test.

Given at 12 mg/kg, i.v. administration of METH elicited a 25% reduction in survival rate at 240 min, which increased to 50% at a dose of 24 mg/kg (Figure 2). Similarly, METH (12 or 24 mg/kg, i.v.) induced a dose-dependent reduction in survival time (Figure 2).

### Depressed brain stem cardiovascular regulation in rats that survived METH intoxication

**Figure 3:**
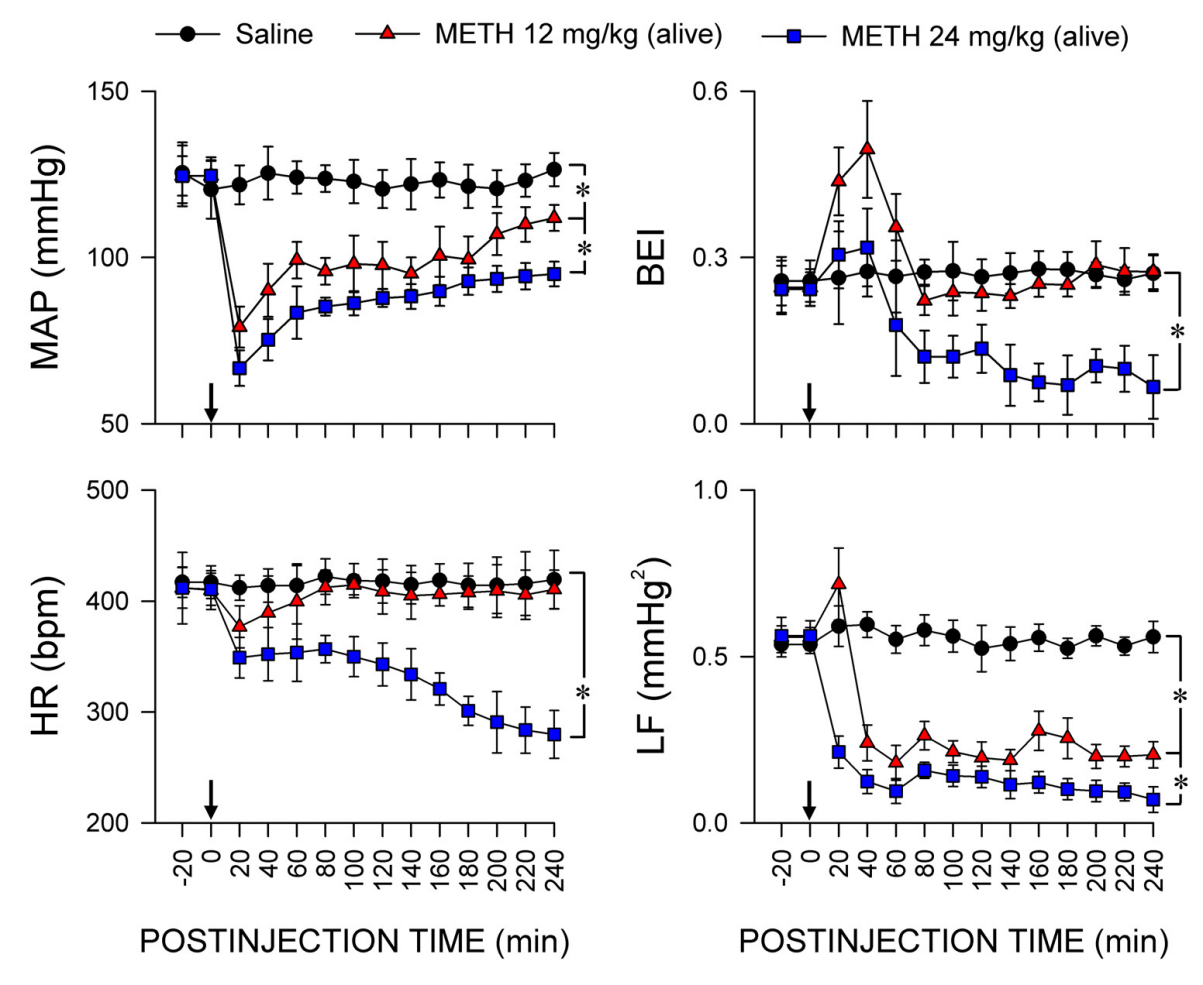
**Depressed brain stem cardiovascular regulation in rats that survived METH intoxication**. Temporal changes in averaged values of mean arterial pressure (MAP), heart rate (HR), baroreflex effectiveness index (BEI) or power density of the low-frequency (LF) component of systolic blood pressure at 20 min intervals in rats that survived intravenous administration of METH (12 or 24 mg/kg; at arrow) or their saline (Sal) controls. Values are mean ± SEM, n = 4-12 animals per experimental group. Note that whereas statistical difference (*P *< 0.05) versus saline group exists in the post hoc Scheffé multiple-range test at corresponding time-points, a single notation (*) is provided for clarity.

The observation that METH manifested the highest concentration in RVLM, CVLM and NTS and the lowest concentration in heart among all tissues studied on systemic administration implies that this psychostimulant may effect circulatory depression not directly on the heart but indirectly via an action on the brain stem cardiovascular regulatory process. Our third series of experiments evaluated this possibility. In animals that survived METH (12 or 24 mg/kg, i.v.), there was a significant and dose-dependent drop in MAP that peaked at 20 min, followed by a gradual return to baseline (Figure 3). HR was essentially maintained in rats that received the lower dose of METH, despite an initial drop at 20 min. On the other hand, there was progressive bradycardia in animals that received the higher dose of METH, with a sustained HR at 200-240 min (Figure 3). Concurrent evaluation of BEI showed an initial augmentation that peaked at 40 min after the administration of METH (12 or 24 mg/kg, i.v.). Whereas BEI returned to baseline in animals that received the lower dose, it underwent a graduate reduction that stabilized at 180-240 min after receiving the higher dose (Figure 3). On the other hand, with the exception of a transient increase at 20 min after administration of METH at 12 mg/kg, both doses elicited a progressive decrease in the power density of LF component of SBP signals over the first 60 min. The LF power, however, lingered subsequently at a low level over the next 120 min (Figure 3).

### Defunct brain stem cardiovascular regulation in rats that succumbed to METH intoxication

**Figure 4:**
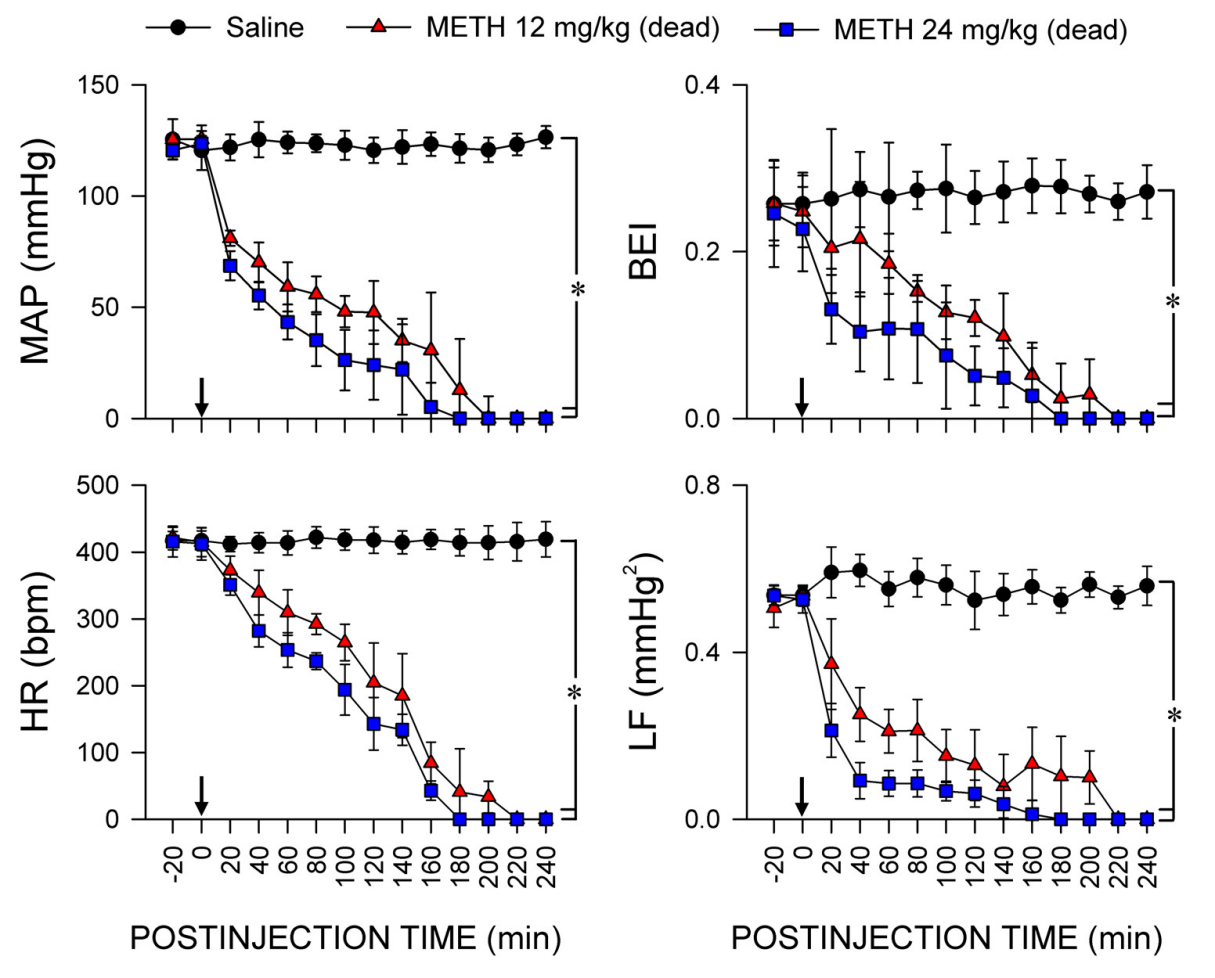
**Defunct brain stem cardiovascular regulation in rats that succumbed to METH intoxication**. Temporal changes in averaged values of MAP, HR, BEI or power density of the LF component of systolic blood pressure at 20 min intervals in rats that succumbed to intravenous administration of METH (12 or 24 mg/kg; at arrow) or in their saline (Sal) controls.. Values are mean ± SEM, n = 4-5 animals per experimental group. Note that whereas statistical difference (*P *< 0.05) versus saline group exists in the post hoc Scheffé multiple-range test at corresponding time-points, a single notation (*) is provided for clarity.

A drastically different picture was revealed in animals that succumbed to METH within 240 min after administration (Figure 4). The initial drop in MAP or HR during the first 20 min seen in animals that survived was followed by a continuous reduction that reached zero 180-220 min after METH administration (12 or 24 mg/kg, i.v.). The initial augmentation of BEI gave way to immediate reduction that became zero at 180 (24 mg/kg) or 220 (12 mg/kg) min. Likewise, the LF power underwent an abrupt decrease and disappeared at 180-220 min. Of note is that the time course of disappearance of MAP, HR, BEI or power density of LF component of SBP signals (Figure 4) was commensurate with the survival time (Figure 2) induced by METH (12 or 24 mg/kg, i.v.).

### Fatality from METH intoxication correlates with its distribution in brain

**Figure 5:**
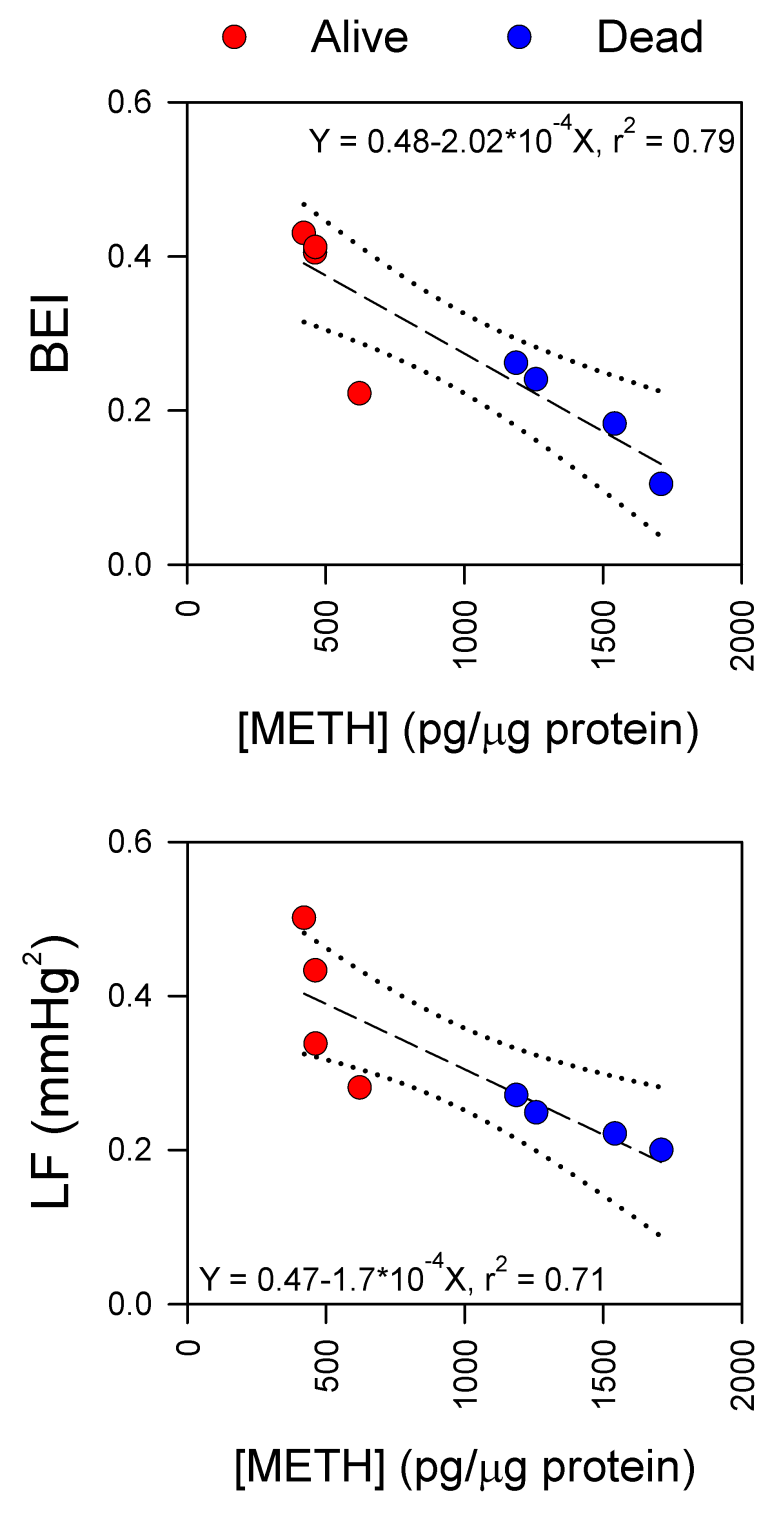
**Fatality from METH intoxication correlates with its distribution in brain**. Linear regression analysis of the relationship between concentration of METH in RVLM and BEI or power density of the LF component of systolic blood pressure. Dotted lines denote 95% confidence intervals, and r^2 ^denotes coefficient of correlation.

Our final series of experiments addressed the issue of whether depressed or defunct brain stem cardiovascular regulation, which dictates survival or death from METH, depends on the distribution of METH to brain stem nuclei that are associated with this homeostatic machinery. We found that the concentration of METH in RVLM, a key neural substrate in brain stem cardiovascular regulation, was positively and significantly correlated with the reduction in BEI and power density of LF component of SBP signals (Figure 5).

## Discussion

The present study revealed two pieces of novel information regarding METH. First, on intravenous administration, this psychostimulant exhibited the largest distribution to brain stem nuclei that are associated with the baroreflex loop, followed by brain targets that are involved in the manifestation of its neurological and psychological responses; the concentration of METH in cardiac tissues was the lowest among all tissues studied. Second, METH effects acute cardiovascular depression by dampening the functional integrity of baroreflex, the degree of which correlated directly with its concentration in the relevant brain stem nuclei.

We chose to employ intravenous administration of METH in the present study because this is a common route [31] that is employed by abusers to rapidly raise the blood concentration of METH to euphoric levels. METH is more lipid-soluble than its primary metabolite amphetamine, with enhanced transport across the blood-brain barrier and stability against enzymatic degradation by monoamine oxidase [32]. Whereas the dose-dependent and time-dependent pharmacokinetics data are in accordance with its chemical properties, we found that instead of NACC, GPi, Cd, SN and VTA, which are involved in the manifestation of its well-known pharmacological effects, the highest concentration of METH was present in RVLM, CVLM and NTS, brain stem nuclei that are associated with the baroreflex circuitry. The ability to maintain a stable AP and HR in the form of baroreflex is essential to humans and animals alike. Inputs from primary baroreceptor afferents synapse at NTS. Outputs from NTS promote modulation of vagal and sympathetic outflow to the heart and peripheral vasculature via the CVLM and RVLM [20]. It is therefore intriguing that the preferential distribution of METH bears functional implications to METH intoxication. In animals that succumbed to METH, the baroreflex-mediated sympathetic vasomotor tone (LF power) and cardiac response (BEI) failed to function (defunct brain stem cardiovascular regulation), leading to the cessation of AP and HR. On the other hand, the power density of LF component of SBP signals and BEI, which either returned to baseline or decreased but stabilized above zero (depressed brain stem cardiovascular regulation) in animals that survived the two doses of METH studied, were sufficient to sustain AP and HR.

We recognize that METH may also act directly on the heart when administered intravenously, leading to heart failure in patients died of METH [1,29,30]. Whereas the design of our study does not allow us to repudiate this notion, the possibility for depression of cardiac functions, rather than failure of brain stem cardiovascular regulation, to be primarily responsible for METH-induced cardiovascular collapse is discernibly reduced by our observation that the distribution of METH to cardiac tissues was significantly less than to brain targets. Furthermore, successful resuscitation of an arrested heart depends on maintained functionality of brain stem cardiovascular regulation [19], suggesting that this homeostatic process, rather than the heart, holds the key to cardiovascular collapse.

METH abusers have to constantly increase dosing to sustain an elevated mood and libido and a decrease in appetite and fatigue. At the same time, continuous use at larger doses results in not only acute METH poisoning but also METH-induced sudden death [16,29,30]. Our novel pharmacokinetics findings offer a reasonable mechanistic underpinning for this phenomenon. We found that the extent that brain stem cardiovascular regulation is dampened is dependent on the concentration of METH in brain stem sites associated with this homeostatic process. An important ramification that arises from this observation is that accompanying the increase in dosing of METH and the sustained euphoric effects is the enhanced possibility for the depressed brain stem cardiovascular regulation to become defunct, leading to cardiovascular collapse. Since METH was determined in cell lysate extracted from the tissue samples, it is possible that our measurements simply represent accumulation of METH and bear no functional relevance. This possibility is deemed unlikely for at least two reasons. First, we demonstrated that the concentration of METH measured in RVLM, a key neural substrate in brain stem cardiovascular regulation, was positively and significantly correlated with the reduction in BEI and power density of LF component of SBP signals. Second, recent studies from our laboratory [33] indicated that the concentration of METH in the extracellular fluid collected by microdialysis from RVLM 10 min after intravenous administration is already 10 times higher than that in serum, approaching a plateau within 20 min.

A majority of the literature on METH concerns primarily with behavioral alterations, physical dependence or psychopathology of withdrawal syndrome and its management that is associated with this psychostimulant. Much less studies are devoted to the mechanisms of cardiovascular collapse associated with METH intoxication despite that it is a common cause of death within the abuse population, [14,15]. The present study revealed that in addition to the brain targets that are associated with the reward system and neurodegenerative diseases, METH exhibits a preferential distribution to brain stem nuclei that are associated with cardiovascular regulation on intravenous administration. We further found that the concentration of METH in those brain stem sites dictates the extent that baroreflex-mediated sympathetic vasomotor tone and cardiac responses are compromised, which in turn determines survival or fatality because of cardiovascular collapse. This information should offer novel leads for devising clinical management or developing therapeutic strategies against the increasing number of sudden death from METH abusers.

## Conclusion

In conclusion, the present study revealed that by exhibiting the largest distribution to brain stem nuclei that are associated with the baroreflex loop on intravenous administration, METH effects acute cardiovascular depression by dampening the functional integrity of this fundamental homeostatic mechanism in brain stem cardiovascular regulation.

## Competing interests

The authors declare that they have no competing interests.

## Authors' contributions

FCHL performed the experiments. JCY participated in experimental design. SHHC and AYWC conceived the study, participated in experimental design, and drafted and revised the manuscript. All authors have read and approved the final manuscript.
